# Automated diagnosis and prognosis of COVID-19 pneumonia from initial ER chest X-rays using deep learning

**DOI:** 10.1186/s12879-022-07617-7

**Published:** 2022-07-21

**Authors:** Jordan H. Chamberlin, Gilberto Aquino, Sophia Nance, Andrew Wortham, Nathan Leaphart, Namrata Paladugu, Sean Brady, Henry Baird, Matthew Fiegel, Logan Fitzpatrick, Madison Kocher, Florin Ghesu, Awais Mansoor, Philipp Hoelzer, Mathis Zimmermann, W. Ennis James, D. Jameson Dennis, Brian A. Houston, Ismail M. Kabakus, Dhiraj Baruah, U. Joseph Schoepf, Jeremy R. Burt

**Affiliations:** 1 Department of Radiology and Radiologic Sciences, Division of Cardiothoracic Radiology, Medical University of South Carolina, Charleston, SC USA; 2 Siemens Healthineers, Malvern, PA USA; 3 Department of Internal Medicine, Division of Pulmonary, Critical Care, Allergy & Sleep Medicine, Medical University of South Carolina, Charleston, SC USA; 4 Department of Internal Medicine, Division of Cardiology, Medical University of South Carolina, Charleston, SC USA; 5 MUSC-ART, Cardiothoracic Imaging, 25 Courtenay Drive, MSC 226, 2nd Floor, Rm 2256, Charleston, SC 29425 USA

**Keywords:** COVID-19, Deep learning, Critical care, Radiology, Pulmonology

## Abstract

**Background:**

Airspace disease as seen on chest X-rays is an important point in triage for patients initially presenting to the emergency department with suspected COVID-19 infection. The purpose of this study is to evaluate a previously trained interpretable deep learning algorithm for the diagnosis and prognosis of COVID-19 pneumonia from chest X-rays obtained in the ED.

**Methods:**

This retrospective study included 2456 (50% RT-PCR positive for COVID-19) adult patients who received both a chest X-ray and SARS-CoV-2 RT-PCR test from January 2020 to March of 2021 in the emergency department at a single U.S. institution. A total of 2000 patients were included as an additional training cohort and 456 patients in the randomized internal holdout testing cohort for a previously trained Siemens AI-Radiology Companion deep learning convolutional neural network algorithm. Three cardiothoracic fellowship-trained radiologists systematically evaluated each chest X-ray and generated an airspace disease area-based severity score which was compared against the same score produced by artificial intelligence. The interobserver agreement, diagnostic accuracy, and predictive capability for inpatient outcomes were assessed. Principal statistical tests used in this study include both univariate and multivariate logistic regression.

**Results:**

Overall ICC was 0.820 (95% CI 0.790–0.840). The diagnostic AUC for SARS-CoV-2 RT-PCR positivity was 0.890 (95% CI 0.861–0.920) for the neural network and 0.936 (95% CI 0.918–0.960) for radiologists. Airspace opacities score by AI alone predicted ICU admission (AUC = 0.870) and mortality (0.829) in all patients. Addition of age and BMI into a multivariate log model improved mortality prediction (AUC = 0.906).

**Conclusion:**

The deep learning algorithm provides an accurate and interpretable assessment of the disease burden in COVID-19 pneumonia on chest radiographs. The reported severity scores correlate with expert assessment and accurately predicts important clinical outcomes. The algorithm contributes additional prognostic information not currently incorporated into patient management.

**Supplementary Information:**

The online version contains supplementary material available at 10.1186/s12879-022-07617-7.

## Introduction

Chest X-rays (CXRs) are important in the initial evaluation of patients with undifferentiated shortness of breath, especially those suspected to have severe acute respiratory syndrome coronavirus 2 (SARS-CoV-2), also known as coronavirus disease 2019 (COVID-19). Advantages of CXRs for suspected COVID-19 include low cost, wide availability, and immediate assessment of disease burden [1]. However, relative quantification of disease extent is subject to interobserver variation, non-specific interpretation, and poorly studied correlations with clinical outcomes. Regardless, for many patients a CXR and nasopharyngeal swab will suffice for the diagnosis of COVID-19 pneumonia, and sometimes a prolonged hospital stay with significant morbidity and mortality will ensue [2].

One potential use for CXRs that is overlooked is the quantitative assessment of disease burden in COVID-19 [3–5]. Radiologists will often comment “bilateral interstitial airspace opacities,” or another qualitative phrase, as the final impression in the report [6]. This overlooks the implication of the distributive burden of airspace disease, which has been investigated previously and is associated with poor outcomes [7, 8]. Certainly, there is more prognostic information which is being left undocumented and may be useful if incorporated into the patient management paradigm [9].

However, quantification of airspace opacity severity (ASOS) is tedious and impractical for the volume and complexity in a contemporary chest radiologist practice. Deep convolutional neural networks (dCNNs) are one option to allow for quantification of ASOS and to aid the radiologist in capitalizing on the missed prognostic value [10–12]. dCNNs applied to this task have achieved high levels of accuracy with COVID-19 diagnostic area under curves (AUCs) ranging from 0.85 to 0.95 [13–16]. Studies involving artificial intelligence (AI) specific to generation of severity scores usually find an excellent correlation between the AI and expert results (r ~ 0.90) [17, 18].

Unfortunately, many AI studies are plagued by low sample-size, unclear origins of training data (including public datasets with poorly annotated images), lack of a real world testing cohort, and absence of follow-up with clinical outcomes [14]. dCNNs are also notorious for having “black box” outputs and a lack of interpretability [19]. Therefore, it is imperative to construct artificial intelligence approaches with the interpreting clinician in mind who wishes to understand the predictors. It is the purpose of this study to evaluate an interpretable dCNN algorithm using CXRs to both diagnose and prognosticate the progression of COVID-19 from a cross-sectional origin in the emergency department with an emphasis on generalizability.

## Methods

### General methods and patient population

This study was performed by retrospective review after approval from the Office of Institutional Research’s institutional review board (IRB). Need for informed consent was waived per retrospective nature of this study. Inclusion criteria in this study was > 18 years of age, presentation to the emergency department, with a documented real-time SARS-CoV-2 reverse transcriptase polymerase chain reaction (RT-PCR) test within 14 days of admission from the dates of January 1st, 2020, to March 15th, 2021. Exclusion criteria consisted of patients < 18 years of age, who had a pediatric-view CXR, lacked a RT-PCR within 14 days, or had insufficient follow-up time for outcomes analysis (defined as < 1 month after admission). Variables collected included basic demographic information (age, sex, ethnicity, body mass index (BMI)), relevant clinical history (history of hypertension (HTN), diabetes, chronic obstructive (COPD) pulmonary disease, etc.), imaging and laboratory identification (exam codes, imaging date, RT-PCR date, image impression), AI results (ASOS), and outcomes data (hospitalization, intensive care unit (ICU) admission, intubation, and all-cause mortality with duration and dates of each event).

**Fig. 1:**
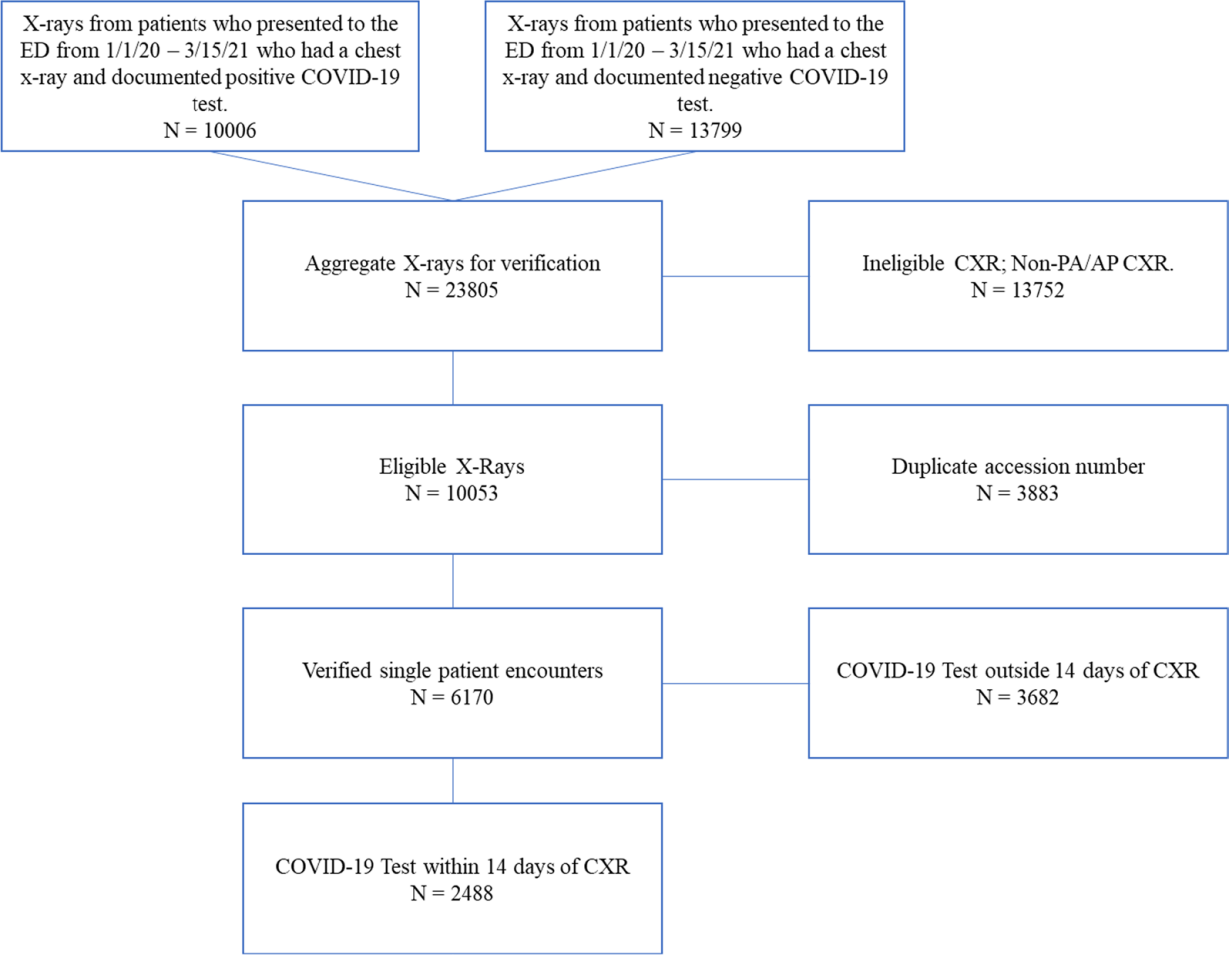
Flow-diagram describing inclusion of patients for COVID-19 training and test datasets. 23,805 X-rays were queried and ultimately 2488 met criteria of a documented COVID-19 test within 14 days of an eligible PA or AP CXR. 2000 were used in the training cohort with 488 retained as internal holdout for validation. Missing data from 32 patients, defined as images that failed the AI segmentation due to poor imaging quality, were excluded

Figure 1 contains a flow diagram describing inclusion of patients for COVID-19 training and test datasets. 23,785 CXRs were queried and ultimately 2456 met criteria of a documented COVID-19 RT-PCR test within 14 days of an eligible PA or AP CXR. A total of 2488 patients were initially enrolled in this study. Missing data from 32 patients, defined as images that failed the AI segmentation due to poor imaging quality, were excluded. The validation cohort consisted of 1000 RT-PCR positive patients and 1000 RT-PCR negative patients. Validation indices include mortality and COVID-19 diagnostic prediction. The test cohort of 456 patients was obtained using a randomized 1:1 internal holdout from the original 2456 patients. Additional file 1: Table S1 contains demographics information for the 2000 training patients.

### Image acquisition and expert evaluation

One-view chest X-rays were obtained according to institutional protocol. Posteroanterior (PA) and anteroposterior (AP) views, but not lateral views, were included in this study. A master list of CXRs for patients who were admitted to the emergency department were obtained via billing code. Images were subsequently exported from the picture archive and communication system without patient identifiers and manually uploaded to Siemens AI-Radiology Companion for evaluation. A total of 2456 images were used in this study. Categorical airspace opacities were defined as presence of airspace disease regardless of severity.

A panel of three fellowship-trained cardiothoracic radiologists independently quantified the airspace opacity severity score for all 2456 images (~ 800 randomized chest radiographs each) for use in ground truth of this study. Briefly, each CXR was evaluated for the presence of pulmonary opacification according to the following [20]:

“The presence of patchy and/or confluent airspace opacity or consolidation in a peripheral and mid to lower lung zone distribution on a chest radiograph obtained in the setting of pandemic COVID-19 was highly suggestive of severe acute respiratory syndrome coronavirus 2 infection…” Airspace opacity severity (ASOS) was determined by visually estimating the percentage of lung involved with airspace opacification. The percentage of lung involvement was then converted into a whole number. For example, if 40% (score = 2/5 or 2) of the right lung and 60% (score = 3/5 or 3) of the left lung contained airspace opacities, the ASOS would be 5 (2 + 3). ASOS ranged from 0 to 10 for each CXR. The score can also be calculated by summing the percentage of airspace opacities in each lung and then multiplying by 0.5.

### Deep convolutional neural network algorithm

The CNN was previously trained on 11,622 cases with 5653 images positive for airspace opacities. Additionally, a set of 540 cases (261 positives for airspace opacities) was previously used as validation and for initial model selection. This patient cohort consisted of adult patients with a mix of typical and atypical infectious pneumonia and was trained to recognize airspace opacities. The predictive models were then trained on 2000 patients (1000 RT-PCR Positive and 1000 RT-PCR Negative) from this study’s CXR dataset. Analysis on the 2000 additional patients before the test dataset can be found in the supplemental material. The following description is designed to fulfill the Checklist for Artificial Intelligence in Medical Imaging (CLAIM) criteria for reproducibility in machine learning as well as avoiding common pitfalls in COVID-19 machine learning studies [14, 21].

**Fig. 2:**
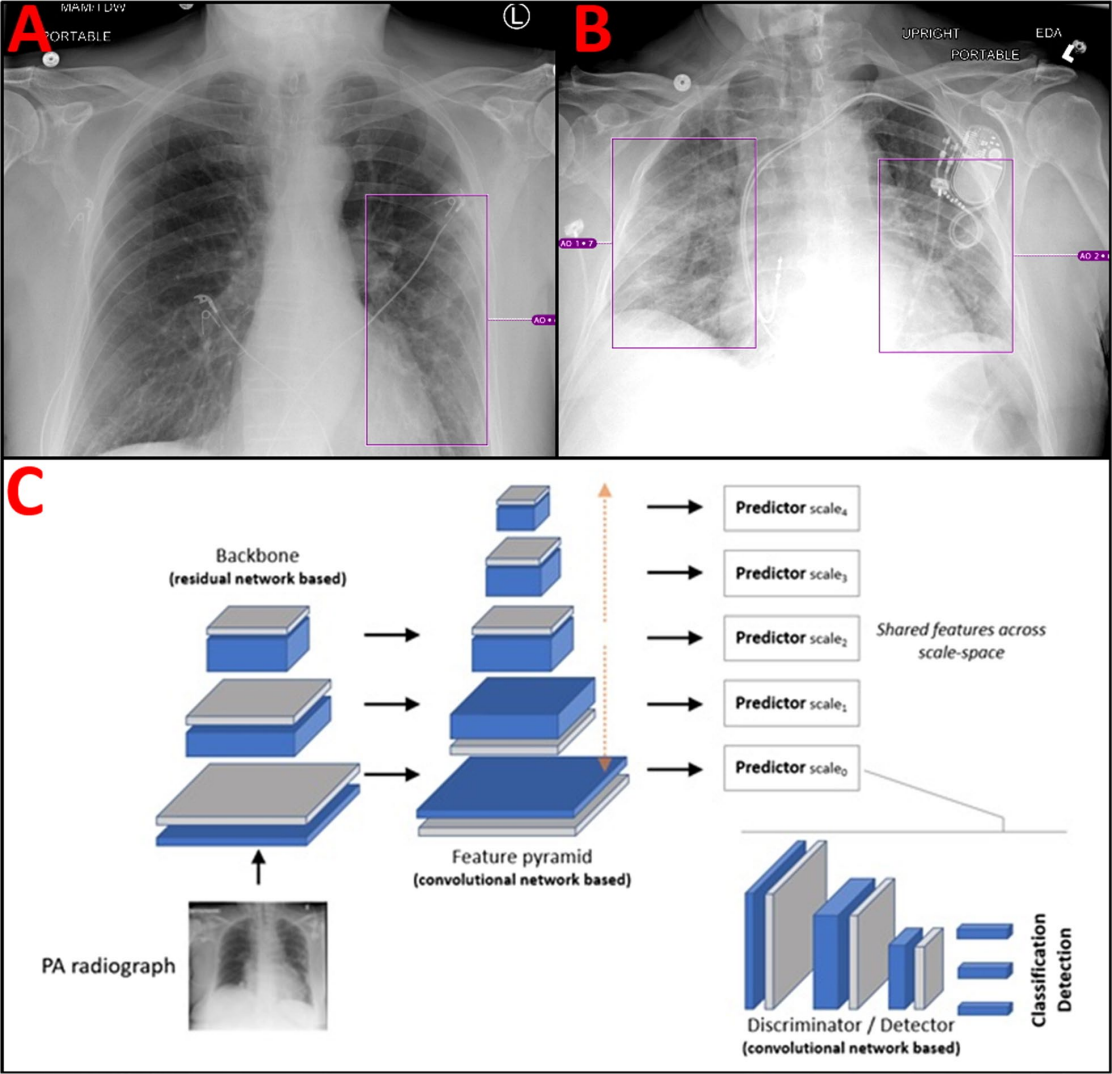
Visual representation of neural network annotations and outputs. **A** AP portable CXR with left lower lobe airspace opacities scored a 4/10 by the dCNN. EKG leads overlie the chest bilaterally. **B** Upright portable AP view CXR with bilateral airspace opacities scored an 8/10 by the dCNN. Dual chamber pacemaker with atrial and ventricular leads overlies the left chest. **C** dCNNs architecture used for classification and detection of airspace opacities. A ResNet backbone for the image anatomy feeds forward into a voxel feature pyramid which is then forwarded to a convolutional network-based detector for classification of the airspace opacity. A detailed description of the architecture can be found in the materials and methods under *Deep Convolutional Neural Network Algorithm*

The architecture of the proposed dCNNs model comprises an early feature extractor acting as candidate generator in an abstract feature space, followed by a discriminator sub-network used to compute probabilities on whether the abnormality is present or not (in an image sub-region of interest) [FCOS]. The architecture is fully convolutional and processes the entire image content in one single pass, while analyzing its content on multiple levels of scales. As such, the architecture is capable of implicitly capturing both global as well as local comorbidities present in the image. Severity score was based on a summation of the geographical extent (as represented by the bounding boxes) of airspace opacities present in both lungs converted into a whole number ranging from 0 to 10. Figure 2A gives an example of a CXR with a low-moderate airspace opacity severity score of 4/10 (~ 40%). EKG leads overlie the chest. Figure 2B gives an example of a CXR with large volume bilateral airspace opacities. The AI severity score in this case was 8/10 (~ 80%). A dual chamber pacemaker with atrial and ventricular leads overlies the left chest, highlighting the robustness of the algorithm for patients with overlying chest hardware. Figure 2C describes the dCNN architecture used in this study. For full details of the neural network architecture please see Homayounieh et al. 2021 Appendix E from which the architecture is sourced [22].

### Model input and output at inference

The input to the model presented in Fig. 2C was an image rescaled to an isotropic resolution of 1025 × 1025 pixels using letterboxing. The output was a set of boxes indicating the location of the abnormalities (airspace disease), each associated with a label and a probability. As a pre-processing step, the images were rescaled to an isotropic resolution of 1025 × 1025 pixels using letterboxing. Bilinear interpolation was used for resampling, followed by a robust brightness/contrast normalization is performed based on a linear remapping of the pixel values.

Training was conducted in one end-to-end manner. The loss function is based on summation of three elements: (1) a classification loss based on the focal loss described in detail in Tsung-Yi et al. [23]; (2) a bounding box coordinate regression loss based on an intersection-over-union based metric; and (3) a center-ness loss designed to reduce outlier detections which is based on a weighted binary cross entropy loss. A batch-size of 8 was used for training. Separate independent validation set was used for model selection and perform early stopping, if necessary. For augmentation we used various intensity and geometric transformations [23, 24].

### Statistical analysis

A power calculation beforehand was performed for the purpose of prediction of outcomes; assuming a 1:10 ratio of events in a 1:1 case: control split, 429 patients were required for a power of 0.9. Prediction of positive SARS-CoV-2 RT PCR results was established using simple logistic regression. Additional file 1: Fig. S1, Tables S2 and S3 provide the power calculation materials. All simple logistic regression variables were constrained by alpha of 0.05 and measures of model performance included Akaike information criterion (AIC) and pseudo-R^2^ (McFadden). All models were evaluated using receiver-operator characteristic (ROC) curves with area under curve (AUC) with 95% confidence interval as the primary measure of prediction. DeLong’s test of two correlated ROC curves was used for statistical comparison. Extracted logistic probabilities were evaluated from the simple logistic regression models. For multivariate analysis, demographics and clinical variables known to be associated with poor outcomes in COVID-19 from the literature were loaded on the initial regression model. A stepwise-backwards logistic regression model was then applied until all variables remaining were considered significant in the model (P < 0.05). Competing models were evaluated using AIC. Optimal threshold values were empirically determined using bootstrapping. Briefly, 400 bootstrapped 1:1 COVID + /COVID- samples were run and the most accurate values were selected. All statistical analysis was performed in R statistical programming version 3.6.3.

## Results

### Patient characteristics

There were 236 COVID-19 positive patients and 220 COVID-19 negative patients included (total = 456). COVID positive patients were more likely to be obese, have diabetes, be organ transplant recipients, and have chronic kidney disease. There was a relatively even dispersion of sex (52.1% male vs 49.5%). There were fewer White or Caucasian patients amongst the COVID-19 positive group (37.2% vs 51.4%). Instead, there was an increase in percentage of Black or African American and Hispanic or Latino people amongst the positive group (50.2% and 7.6% vs 45.0% and 0%, respectively) (Table 1).

**Table 1:** Demographics and clinical variables of test cohort patients stratified by SARS-CoV-2 RT-PCR results

Variables	RT-PCR Positive (N = 236)		RT-PCR Negative (N = 220)	
*N* = *456*	Mean	SD	Mean	SD
Age (years)	55.3	17	49.2	16.3
BMI kg/m^2^	31.6	8.5	27.7	7.4
CXR–PCR Interval (days)	3.4	3.8	3.1	14.4

	Count	Frequency (%)	Count	Frequency (%)
Sex				
Female	113	47.9	111	49.5
Male	123	52.1	109	50.5
Ethnicity				
Asian	2	0.9	2	0.9
Black	112	47.5	99	45.0
Hispanic	17	7.2	0	0
Other	9	3.8	6	2.7
White	83	35.2	113	51.4
Smoking				
Never	155	65.7	93	42.3
Former	19	8.1	73	33.2
Current	54	22.9	54	24.5
COPD	22	9.3	10	4.5
Cystic fibrosis	1	0.4	0	0
Asthma	32	13.6	35	15.9
Lung cancer	2	0.8	0	0
Cancer (other)	36	19.2	29	13.2
Diabetes mellitus	92	39.0	48	21.8
Hypertension	148	62.3	114	51.8
Cardiac disease	26	11.0	52	23.6
Pulmonary HTN	23	9.7	6	2.7
Sickle cell disease	6	2.5	18	8.2
Thalassemia	0	0	0	0
Organ transplant	13	5.5	4	1.8
HIV	1	0.4	4	1.8
Autoimmune	15	6.4	14	6.4
Chronic liver disease	7	3.0	8	3.6
Chronic kidney disease	48	20.3	17	7.7

SARS-CoV-2: Severe acute respiratory syndrome coronavirus 2; RT-PCR: Reverse transcription polymerase chain reaction; SD: Standard deviation; BMI: Body mass index; CXR: Chest X-ray; COPD: Chronic obstructive pulmonary disease; HTN: Hypertension; HIV: Human immunodeficiency virus

### Agreement and model performance

**Fig. 3:**
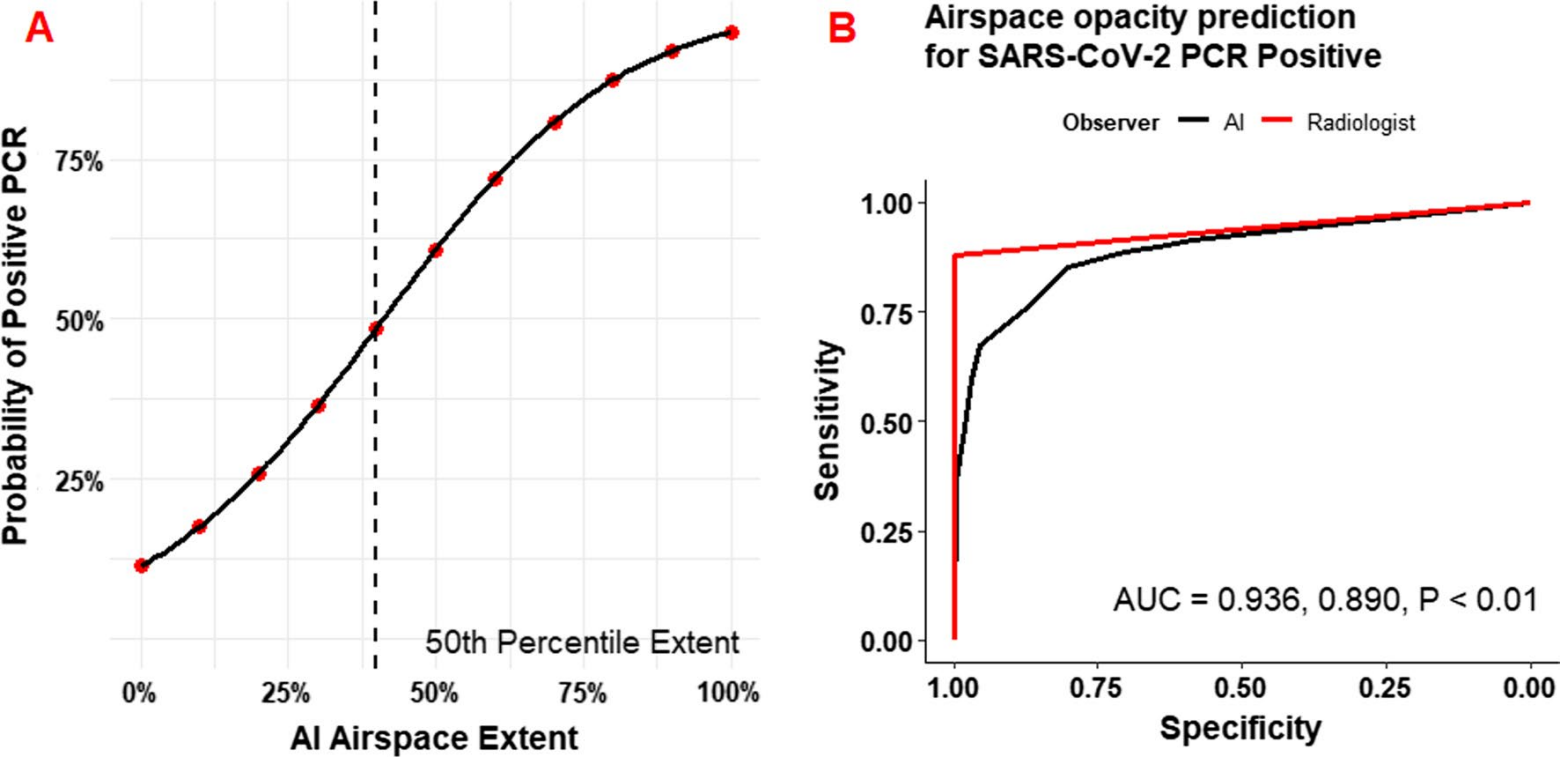
Prediction of Positive SARS-CoV-2 PCR by extent of AI-determined airspace disease. **A** Logistic probability plot of positive SARS-CoV-2 PCR as a function of AI-determined airspace extent. Median airspace extent (40%) had just under 50% probability of a concurrent positive PCR. McFadden R^2^ = 0.412. **B** ROC curve for prediction of SARS-CoV-2 PCR positivity in comparison to radiologist impression of airspace extent. Radiologist (AUC = 0.936, 95% CI 0.918–0.960) and AI (AUC = 0.890, 95% CI 0.861–0.920) annotations were both highly accurate

Figure 3 demonstrates the prediction of SARS-CoV-2 RT-PCR results by AI-determined ASOS (AI-ASOS). The probability of a positive PCR approaches 1 as a logistic function of AI-ASOS. At the median AI-ASOS (40%) there was a ~ 50% probability of a positive result. Radiologist (AUC = 0.936, 95% CI 0.918–0.960) and AI (AUC = 0.890, 95% CI 0.861–0.920) annotations were both highly accurate with a slight advantage for the radiologist measurement (P < 0.01). For comparison, the impressions on the original clinical radiology reports are aggregated and listed in Additional file 1: Tables S4 and S5. The sensitivity of expert reads for a diagnosis of COVID-19 was 88.4% and the sensitivity of the AI for any airspace opacity was 91.5% Ninety nine percent (218/220) of negative nasopharyngeal swabs had corresponding CXRs read as “No evidence of acute cardiopulmonary disease,” while only 45.1% (106/235) of CXRs associated with positive SARS-CoV-2 RT-PCR tests were reported as consistent with COVID-19.

**Fig. 4:**
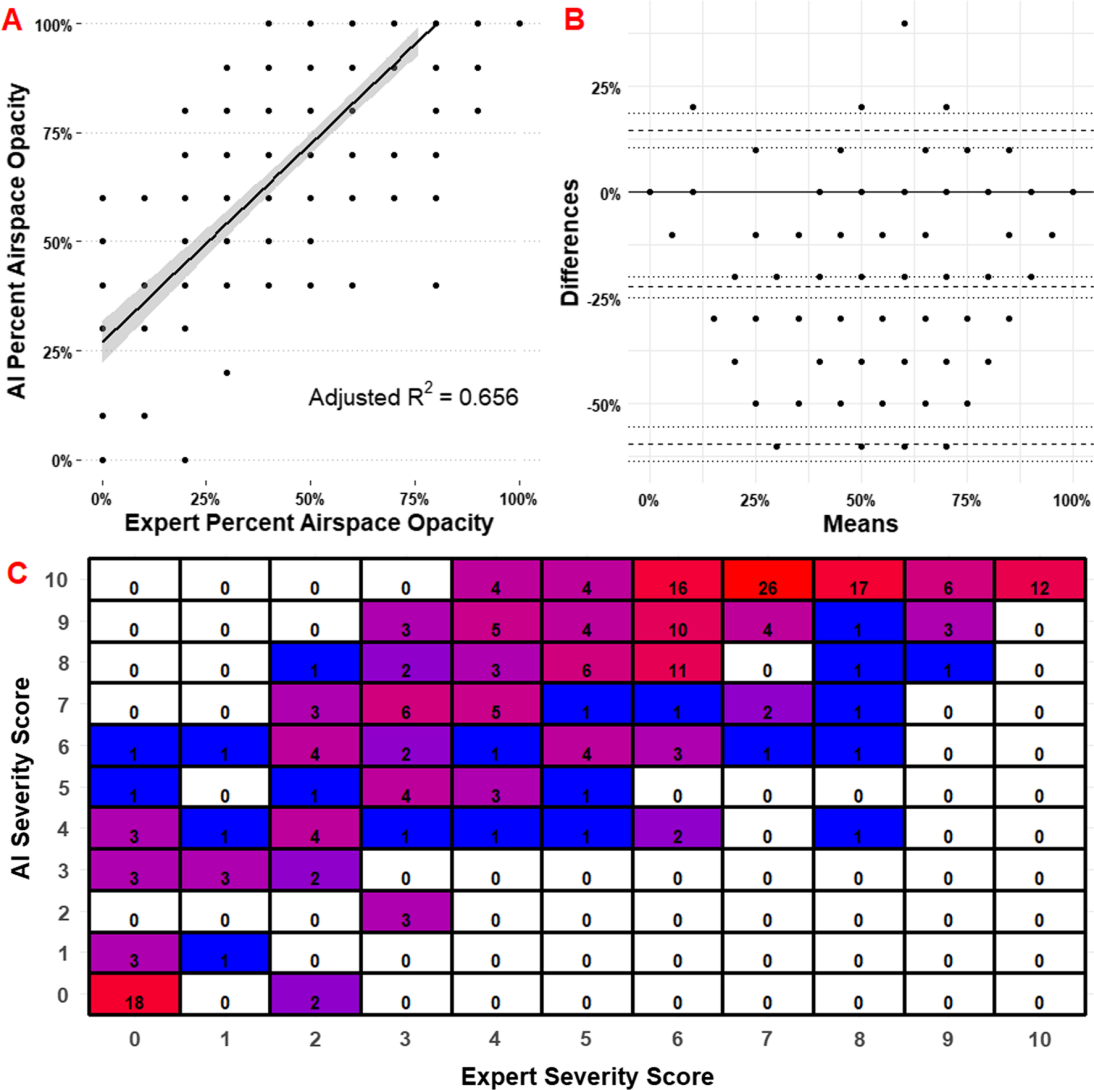
Comparison of differences between AI and Radiologist measurement of airspace opacity extent. **A** Airspace opacity extent percentage as a function of observer. Adjusted R^2^ = 0.656; Spearman ρ = 0.797. Overall agreement is considered excellent for positive cases (single fixed raters ICC = 0.810, 95% CI 0.765–0.840). Agreement for all cases is considered excellent (single fixed raters ICC = 0.820, 95% CI 0.790–0.840). **B** Bland–Altman plot for difference of methods. Mean difference -22.4%; SE 21.1%. **C** Confusion matrix for discrete scores compared between expert and AI. Weighted macro F1 score for categorical agreement is 0.157

Figure 4 describes the interobserver agreement of the AI and radiologists. Figure 4A demonstrates airspace opacity extent percentage as a function of observer. Adjusted R^2^ = 0.656; Spearman ρ = 0.797. Overall agreement is considered excellent for positive cases (single fixed raters ICC = 0.810, 95% CI 0.765–0.840). Agreement for all cases is considered excellent (single fixed raters ICC = 0.820, 95% CI 0.790–0.840). Figure 4B contains comparison of differences by Bland–Altman plot. Mean difference − 22.4%; SE 21.1%. Additional file 1: Table S4 contains the qualitative analysis of concordance and accuracy. Radiologists had an accuracy of 0.936 (95% CI 0.910–0.960) and AI had an accuracy of 0.757 (95% CI 0.715–0.795) for the detection of any lesion. AI sensitivity (0.915, 95% CI 0.872–0.947) was near radiologist sensitivity (0.884, 95% CI 0.835–0.919). Cohen’s Kappa for radiologists and AI versus RT-PCR was 0.873 and 0.507, respectively. Categorical contingency data reveals a bias for AI to overestimate the severity of illness.

Table 2 contains the diagnostic thresholds for the most accurate, most sensitive, and most specific models (40%, 10%, and 80%, respectively). An AI-ASOS of > 40% had accuracy of 81.8% (95% CI 0.783–0.853) for a positive RT-PCR test. > 10% had a sensitivity of 0.898 (95% CI 0.852–0.934) and > 80% had a specificity of 0.968 (0.936–0.987). The odds ratio for a positive RT-PCR test amongst patients with > 40% severity was 20.9 (95% CI 12.9–33.7). Additional file 1: Fig. S2 contains the rationale for empiric derivation of interpretable AI-ASOS cutoffs for SARS-CoV-2 RT-PCR results. The most accurate AI-ASOS values falls between 40 and 50%.

**Table 2:** Diagnostic performance of empirically derived threshold models for SARS-CoV-2 RT-PCR Positivity

	Accuracy	Sensitivity	Specificity	PPV	NPV
Metric					
** ≥ 40%**	**0.818 (0.783**–**0.853)**	0.792 (0.735–0.842)	0.850 (0.791–0.891)	0.850 (0.799–0.894)	0.792 (0.740–0.843)
> 10%	0.776 (0.738–0.815)	**0.898 (0.852**–**0.934)**	0.646 (0.578–0.709)	0.731 (0.680–0.782)	**0.855 (0.802**–**0.909)**
> 80%	0.774 (0.736–0.813)	0.593 (0.528–0.656)	**0.968 (0.936**–**0.987)**	**0.952 (0.918**–**0.987)**	0.689 (0.638–0.741)
Most accurate model (AI airspace opacity severity ≥ 40%)					
False Positive Rate	0.155 (0.107–0.202)	LR+	5.13 (3.74–7.03)	RR	4.06 (3.15–5.23)
False Negative Rate	0.208 (0.156–0.259)	LR−	0.246 (0.190–0.317)	OR	20.9 (12.9–33.7)

Bolded values indicate highest values for each category

SARS-CoV-2: severe acute respiratory syndrome coronavirus 2; RT-PCR: reverse transcription polymerase chain reaction; PPV: positive predictive value; NPV: negative predictive value; LR: likelihood ratio; RR: relative risk; OR: odds ratio

### Prediction of outcomes

Table 3 contains the univariate outcomes analysis stratified amongst SARS-CoV-2 PCR results. Higher ASOS was differentially associated with all measured outcomes between COVID-19 and control patients (P < 0.001 for hospitalization, ICU admission, intubation, ARDS, mortality, and pulmonary mortality). Mean ASOS increased sequentially in terms of outcome severity (µ-hospitalization = 5.4 (SD 4.0), µ-ICU admission = 8.3 (SD 2.5), µ-mortality = 8.6 (SD 2.2).

**Table 3:** Association of AI-ASOS with clinical outcomes amongst patients stratified by SARS-CoV-2 RT-PCR

Outcome	N	SARS-CoV-2 (+)		N	SARS-CoV-2 (−)		P
		Mean ASOS	SD		Mean ASOS	SD	
Hospitalization	175	5.4	4.0	124	2.7	3.3	< 0.001
ICU admit	120	8.3	2.5	10	3.0	3.4	< 0.001
Intubation	88	7.7	3.3	17	3.6	3.7	< 0.001
ARDS	115	8.8	1.9	1	3.0	3.4	< 0.001
Mortality	53	8.6	2.2	2	3.9	3.8	< 0.001
Pulmonary mortality	47	8.9	1.9	0	–-	–-	–

SARS-CoV-2: Severe acute respiratory syndrome coronavirus 2; RT-PCR: Reverse transcription polymerase chain reaction; ASOS: Airspace Opacity Severity Score; SD: Standard deviation; ICU: Intensive care unit; ARDS: acute respiratory distress syndrome

**Fig. 5:**
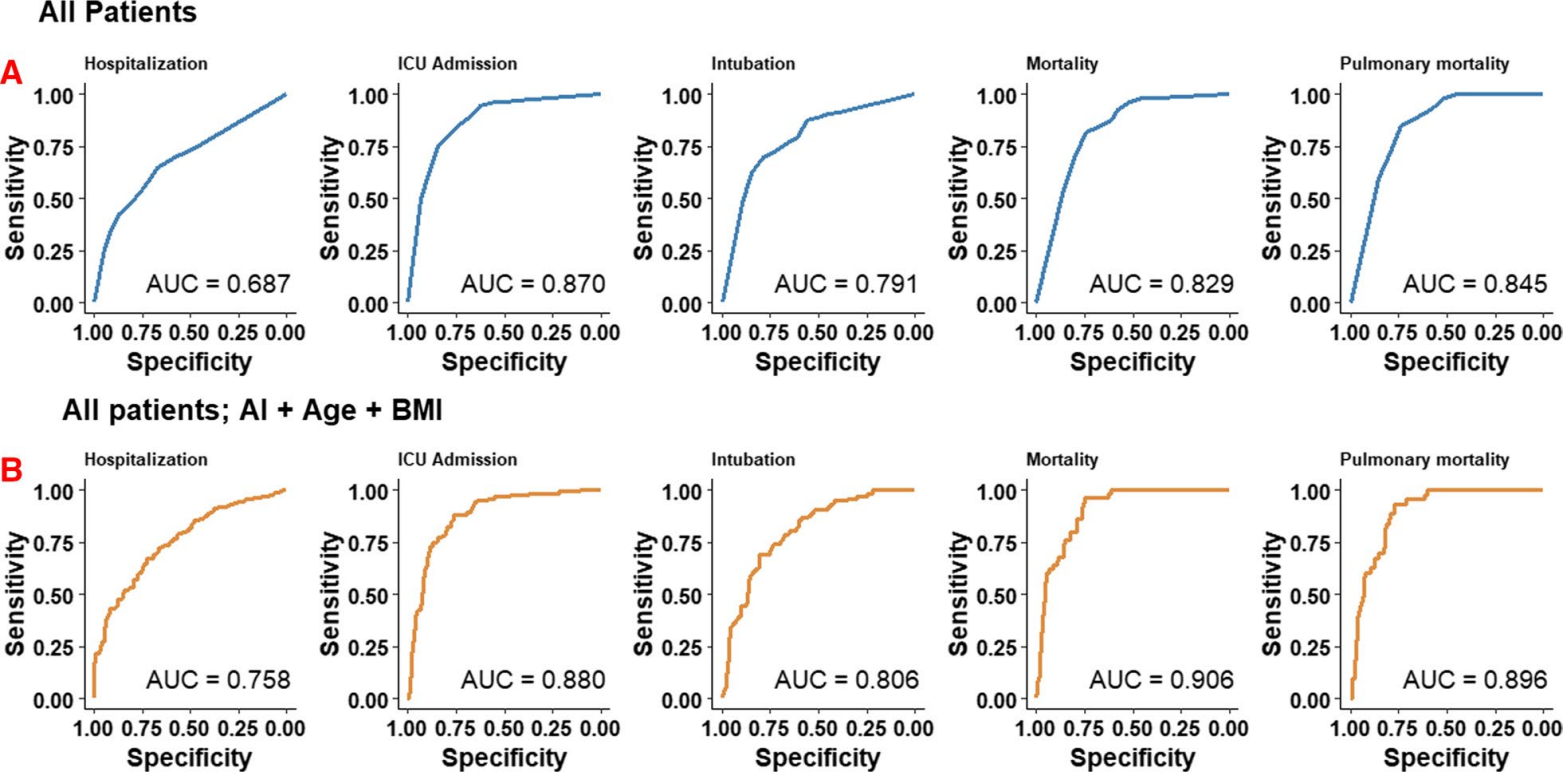
Prediction of outcomes by use of AI-determined airspace opacity extent (AI-ASOS) using simple logistic regression. **A** Prediction of outcomes in all patients. AI-ASOS is best at predicting ICU admission (AUC = 0.870, 95% CI 0.834–0.904) and pulmonary mortality (AUC = 0.845, 95% CI 0.802–0.888). **B** Prediction of outcomes statistics amongst all patients using a multivariate empirically derived model of additional clinical risk factors. Use of AI-ASOS, age, and BMI had a high accuracy for prediction of mortality statistics and ICU admission (AUC = 0.906, 0.896, and 0.880, respectively)

Figure 5 contains the logistic regression model predictions of outcomes stratified across all patients (5A) and all patients in a multivariate model with age and BMI (5B). AI-derived ASOS as a single factor highly predicted ICU admission, intubation, and mortality in all patients upon initial ER presentation (AUC = 0.870, 0.791, and 0.829, respectively). Addition of age and BMI in a multivariate logistic regression model resulted in modest improvements in overall predictive scores. Multivariate prediction of mortality increased from 0.829 to 0.906. Integer increases in odds ratios of listed outcomes range from 1.2 to 1.59 (Table 4).

**Table 4:** Logistic regression model parameters and predictive intervals for AI severity scores alone and with age + BMI

	McFadden R^2^	OR Score (95% CI)	AUC (95% CI)
*AI Score Alone*			
Hospitalization	0.082	1.20 (1.14–1.27)	0.687 (0.639–0.735)
ICU admission	0.336	1.57 (1.45–1.72)	0.869 (0.834–0.934)
Intubation	0.186	1.36 (1.26–1.46)	0.791 (0.742–0.840)
Mortality	0.226	1.51 (1.34–1.73)	0.829 (0.782–0.876)
Pulmonary mortality	0.244	1.59 (1.39–1.90)	0.845 (0.802–0.888)
*AI Score* + *Age* + *BMI*			
Hospitalization	0.153	1.22 (1.14–1.31)	0.758 (0.710–0.806)
ICU admission	0.359	1.59 (1.45–1.75)	0.880 (0.845–0.915)
Intubation	0.202	1.36 (1.26–1.48)	0.806 (0.759–0.853)
Mortality	0.369	1.55 (1.35–1.84)	0.906 (0.873–0.939)
Pulmonary mortality	0.331	1.55 (1.33–1.85)	0.896 (0.860–0.932)

AI: Artificial Intelligence; BMI: Body Mass Index (kg/m^2^); OR: Odds ratio; AUC: Area under curve; CI: Confidence Interval; ICU: Intensive care unit

**Fig. 6:**
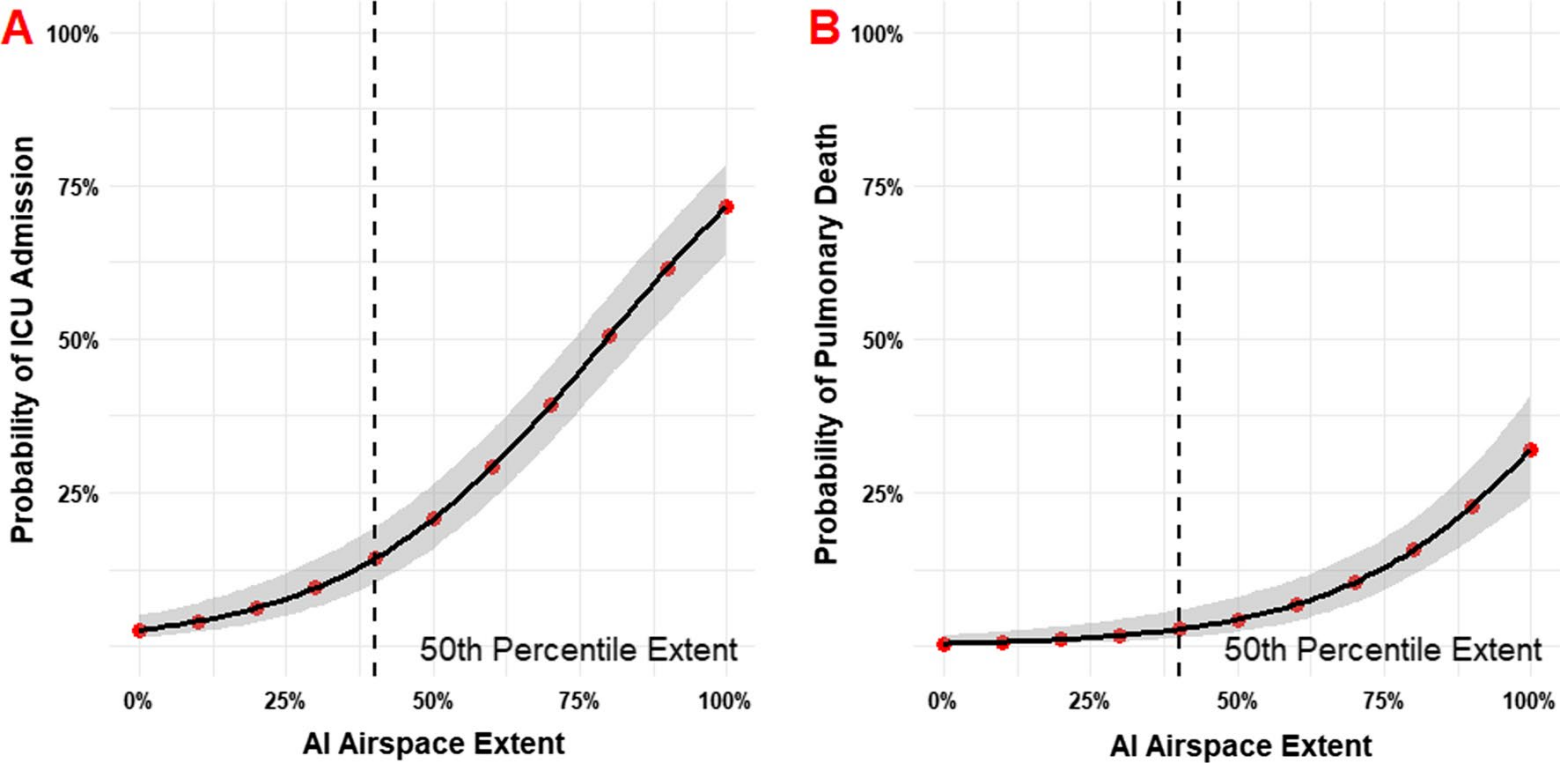
Probabilities of outcomes as a function of AI-determined airspace opacity extent (AI-ASOS). **A** Probability of ICU admission. 50% airspace opacity extent (AI-ASOS = 5) confers a ~ 20% chance of ICU admission. **B** Probability of pulmonary death. Risk of pulmonary death begins increasing at roughly 50% airspace opacity extent (AI-ASOS = 5)

Figure 6 demonstrates the probability of ICU admission and subsequent pulmonary-related mortality as a function of AI-derived ASOS at initial presentation to the ER. The 50th percentile AI-ASOS corresponded with ~ 12.5% probability of ICU admission and < 10% risk of pulmonary mortality. A 75% AI-ASOS was associated with roughly a 50% probability of ICU admission and 12.5% risk of mortality. 100% AI-ASOS was associated with an ICU admission probability of nearly 75% and mortality of > 25%.

## Discussion

This study was performed to evaluate an interpretable dCNN algorithm using CXRs to both diagnose and prognosticate COVID-19 disease from patients initially presenting to the emergency department with possible COVID-19 symptoms at a single institution. The prognostication of COVID-19 on CXR currently is not well quantified. Quantification of airspace opacities is tedious and difficult to perform at volume but yields valuable prognostic information [9]. Automating quantitative and repetitive tasks is where deep learning excels, but to implement clinically requires understanding of the predictors and relevant clinical interpretability of the results for both the ordering clinician and the radiologist [19, 25].

The relevance of chest radiography for the evaluation of COVID-19 pneumonia is well established and conforms to existing American College of Radiology appropriate use guidelines for patients with acute respiratory complaints [26]. Briefly, the Fleischner society of thoracic radiology highlights the indication of chest imaging for COVID-19 patients in a 2020 white paper:

“For COVID-19 positive patients, imaging establishes baseline pulmonary status and identifies underlying cardiopulmonary abnormalities that may facilitate risk stratification for clinical worsening… CXR can be useful for assessing disease progression and alternative diagnoses such as lobar pneumonia, suggestive of bacterial superinfection, pneumothorax, and pleural effusion…” [27].

In this study we demonstrated a highly accurate and interpretable deep learning algorithm for diagnosis of COVID-19 on chest radiographs that approaches expert discrimination. Most importantly, the quantification of airspace opacities had a high degree of reliability with high sensitivity. Several important diagnostic and inpatient prognostic heuristics were identified. AI-derived ASOS as a single factor highly predicted ICU admission, intubation, and mortality in all patients upon presentation (AUC = 0.870, 0.791, and 0.829, respectively). Finally, addition of age and BMI increased the AUC of mortality from 0.829 to 0.906.

Amongst many clinicians, deep learning has developed a reputation for being a “black box” with mysterious derivation of clinical utility [28]. It is important for all parties to be able to interpret the data at hand, from the ordering provider in the ED or floor to the patient and their family in the ICU discussing goals of care and probability of significant events. In this study we show that a deep learning model can be applied to provide interpretable, actionable prognostic information regarding the disease course and progression of COVID-19. Added value over current protocol is derived from the quantification of airspace opacities, which is currently not standard of practice for expert chest radiologists.

There are many published examples of the application of deep learning and pre-trained neural networks to the assessment of COVID-19 on plain films. A variety of approaches have been taken, most notably involving ResNet/U-Net and other publicly available architectures (ResNet50, ResNet101, ResNet150, InceptionV3 and Inception-ResNetV2, etc.). These available architectures have been reported to approach accuracies as high as 99% but perform less optimally with the introduction of more complex tasks [29]. A recent article found accuracies ranging from 82 to 99% for the binary classification of normal vs COVID-19 pneumonia amongst a wide range of models. The authors of the mentioned study proposing a hybrid model with accuracy reaching 99.05%, near identical to nasopharyngeal RT-PCR [30, 31]. The baseline accuracy in this study was found to be 89% for the AI and 93% for the radiologist, comfortably within the range of other reported values in the literature. Given the high ICC (0.820), the authors conclude the AI nearly approximates expert scoring; further modification is needed to truly approach inter-expert reliability (0.9–0.95).

The AI-quantified airspace opacities predict hospitalization, ICU admission, intubation, and death along with the probability of these events as a function of time. Implications include accurate evaluation of need for advanced level of care. For instance, a patient with a severity score of 7–8 has a 50% probability of ICU admission in this study. Utilization of the AI algorithm at a facility with capped or limited ICU structure could alert the institution to seek escalation in level of care from as early as presentation to the emergency department. For clinicians on the floor evaluating a patient with deteriorating respiratory status, the clinician would be able to utilize the probability of intubation and death in discussion of goals of care upon admission to the ICU. Both patients and clinicians would benefit from having probabilistic information available to enhance shared decision making. Incorporation of other clinical factors such as age and BMI only enhance the predictive capabilities, leading to adjustment for individual clinical situations.

The practical applications of the AI software to calculate airspace opacity scores would be as an adjunct order for radiologists or clinicians at the point of care. Radiologists or radiology technologists could apply the AI algorithm beforehand from a compatible workstation when the ordering indication contains COVID-19, during the interpretation when the radiologist deems the most likely diagnosis to be COVID-19 pneumonia, or afterwards when the ordering clinician wishes to contextualize the findings in terms of patient hospitalization trajectory. These triggers could be automated according to institutional protocol and preferences and do not necessarily need to be applied to all patients.

Limitations of this study include the retrospective nature of the test cohort and the singular use of emergency department plain films without a lateral view that decreases generalizability of the findings to only the use cases presented. This study also does not evaluate changes associated with serial imaging or evolving clinical situations. Further study is needed to evaluate the changes in serial CXRs and the relationship between ASOS and deteriorating clinical status. This study also lacks a true external testing cohort. Further study should be multicenter, randomized, and prospective to improve generalizability. Finally, this study also makes no reference to the individual strains of COVID-19 or vaccination status, as enrollment concluded before the preponderance of the delta variant or widespread vaccination. Adjustment for these factors may contribute to more accurate prognostication and generalizability of the model.

## Conclusions

The AI was developed to evaluate CXRs to both diagnose and prognosticate COVID-19 disease from patients initially presenting to the emergency department with possible COVID-19 symptoms. Our findings support that this AI algorithm is highly accurate and approaches cardiothoracic radiologist performance. The airspace opacity severity score produced by the AI model is highly related to the incidence of clinically important outcomes and provides additional prognostic information that is not currently part of the standard of practice.

## Supplementary Information

**Additional file 1.** Additional figures and tables.

## Data Availability

The availability of study data including clinical demographics and outcomes is not publicly available to protect patient privacy, but the data may be released upon reasonable request to the corresponding author. MUSC used a Siemens prototype of the software, which was delivered to MUSC under a contract and Master Research Agreement and was only for use at MUSC for a limited time. Unfortunately, the algorithm cannot be shared publicly. The raw image dataset generated or analyzed during this study is not publicly available due to the DICOM metadata containing information that could compromise patient privacy/consent. Radiologic images used in this article are completely deidentified, and no details are reported on individuals within the manuscript.

## Abbreviations

AI: Artificial intelligence
AIC: Akaike information criterion
AP: Anteroposterior (view)
ARDS: Acute respiratory distress syndrome
ASOS: Airspace opacity severity
AI-ASOS: Artificial intelligence-derived airspace opacity severity score
AUC: Area under curve
BMI: Body mass index
CI: Confidence interval
COPD: Chronic obstructive pulmonary disease
COVID-19: Coronavirus disease of 2019
CXRs: Chest X-rays
dCNNs: Deep convolutional neural networks
ED: Emergency department
HIV: Human immunodeficiency virus
HTN: Hypertension
ICC: Intraclass correlation coefficient
ICU: Intensive care unit
IRB: Institutional review board
LR: Likelihood ratio
NPV: Negative predictive value
OR: Odds ratio
PA: Posteroanterior (view)
PPV: Positive predictive value
ROC: Receiver-operator characteristic
RR: Relative risk
RT-PCR: Reverse transcriptase polymerase chain reaction
SARS-CoV-2: Severe acute respiratory syndrome coronavirus 2
SD: Standard deviation
SE: Standard error
