# Supplementary material for: Automated diagnosis and prognosis of COVID-19 pneumonia from initial ER chest X-rays using deep learning
Source: BMC Infect Dis. 2022 Jul 21;22:637. doi: 10.1186/s12879-022-07617-7 (PMC9301895; doi:10.1186/s12879-022-07617-7)
Supplement: Supplementary file 1 — Additional file 1. Additional figures and tables. [file 12879_2022_7617_MOESM1_ESM.docx]

**Supplemental material for:** **Automated diagnosis and prognosis of COVID-19 pneumonia from initial ER chest x-rays using deep learning**

Chamberlin et al. 2021.

|  | **COVID Positive (N = 1000)** | | **COVID Negative (N = 1000)** | | **P** |
| --- | --- | --- | --- | --- | --- |
|  | **Median** | **IQR** | **Median** | **IQR** |  |
| **Age (years)** | 58 | 42 - 71 | 47.5 | 34 - 61.3 | <0.001 |
| **BMI (Kg/m^2^)** | 30.8 | 26.2 - 36.5 | 27.4 | 23.6 - 32.7 | <0.001 |
| **Date Difference** | 3 | 0 - 7 | 0 | 0 - 2 | NS |
|  | **Count** | **Frequency (%)** | **Count** | **Frequency (%)** |  |
| **Sex**  Female  Male | 547  453 | 54.7  45.3 | 570  430 | 57.0  43.0 | 0.334 |
| **Ethnicity**  Asian  Black  Hispanic  Other  White | 9  560  22  31  377 | 0.9  56.0  2.2  3.1  37.7 | 13  446  23  19  498 | 1.3  44.6  2.3  1.9  49.8 | <0.001 |
| **Impression**  No Acute Findings  Possible Infection  Probable COVID | 412  320  268 | 41.2  32.0  26.8 | 992  7  0 | 99.2  0.7  0 | <0.001 |
| **Deceased** | 225 | 22.5 | 21 | 2.1 | <0.001 |

**Table S1.** Summary data for training set. COVID + patients were significantly older, more obese, disproportionately identified as black, and were 10 times more likely to be deceased at the time of data collection.

| COVID + | COVID - | Power |
| --- | --- | --- |
| 0 | 0 | 0.050 |
| 100 | 100 | 0.270 |
| 200 | 200 | 0.477 |
| 300 | 300 | 0.644 |
| 400 | 400 | 0.767 |
| 500 | 500 | 0.852 |
| 600 | 600 | 0.909 |
| 700 | 700 | 0.945 |
| 800 | 800 | 0.968 |
| 900 | 900 | 0.982 |
| 1000 | 1000 | 0.990 |

**
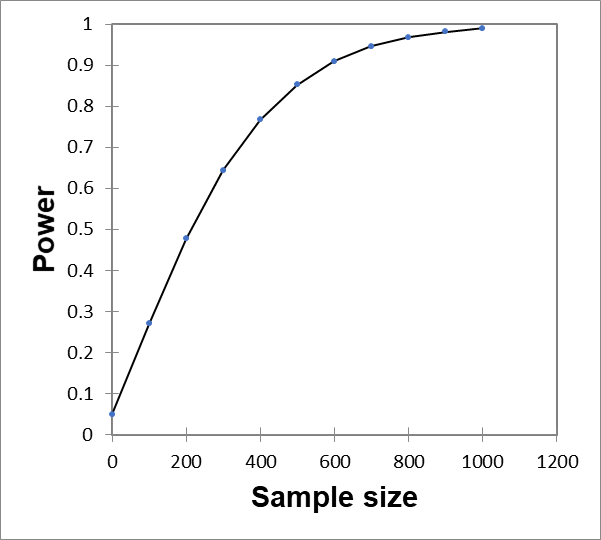
**

**Figure S1.** Using a standard alpha of 0.05, and beta of 0.2 (Power = 0.8), ~ 450 patients in each group are needed for the purpose of comparing the proportion of correct diagnoses within 10% of the truth. Two groups of 1000 give a power of 99%.

| **Power** | **Kappa (N1:N2)** | **Minimum events (case/control)** |
| --- | --- | --- |
| 0.8 | 2 | 37/74 |
| 0.8 | 4 | 32/128 |
| 0.8 | 6 | 31/217 |
| 0.8 | 10 | 29/290 |
| 0.9 | 2 | 48/96 |
| 0.9 | 4 | 42/168 |
| 0.9 | 6 | 40/240 |
| 0.9 | 10 | 39/390 |

**Table S2.** Comparing the AI and Expert ROC curves for predictions of an event. Kappa represents the ratio of non-events to events for a given outcome.

| **N** | **Proportion when x = 0** | **Proportion when x = 1** | **Kappa** | **Power** |
| --- | --- | --- | --- | --- |
| **200** | **0.5** | **0.8** | **2** | **0.995** |
| **200** | **0.5** | **0.8** | **4** | **0.979** |
| **200** | **0.5** | **0.8** | **10** | **0.763** |

**Table S3.** Calculating the power of logistic regression assuming a detectable difference in auc of +/- 0.1 compared to the null hypothesis (no difference).

| Table S4. Qualitative diagnostic accuracy of Radiologists and AI for SARS-CoV-2 RT-PCR Positivity | | | |
| --- | --- | --- | --- |
| Radiologist |  |  | |
|  |  | **PCR Results** | |
|  | **Expert Impression** | Positive | Negative |
|  | Positive | 207 | 0 |
|  | Negative | 29 | 220 |
|  |  |  |  |
| *Accuracy* | 0.936 (0.910 - 0.960) | *PPV* | 1 (0.979 - 1) |
| *Sensitivity* | 0.884 (0.835 - 0.919) | *NPV* | 0.877 (0.827 - 0.915) |
| *Specificity* | 1 (0.977 - 1) | *Kappa* | 0.873 (0.830 - 0.920) |
|  |  |  | |
| Deep Learning |  |  | |
|  |  | **PCR Results** | |
|  | **AI - any opacity** | Positive | Negative |
|  | Positive | 216 | 91 |
|  | Negative | 20 | 129 |
|  |  |  |  |
| *Accuracy* | 0.757 (0.715 - 0.795) | *PPV* | 0.704 (0.652 - 0.755) |
| *Sensitivity* | 0.915 (0.872 - 0.947) | *NPV* | 0.866 (0.811 - 0.921) |
| *Specificity* | 0.586 (0.518 - 0.652) | *Kappa* | 0.507 (0.432 - 0.583) |
| SARS-CoV-2 = Severe acute respiratory syndrome coronavirus 2, RT-PCR = Reverse transcription polymerase chain reaction, AI = Artificial Intelligence, PPV = Positive predictive value, NPV = Negative predictive value, Kappa = Cohen’s Kappa | | | |

**Table S4**. Analysis of expert and deep learning qualitative metrics obtained from exam impressions in the patient chart prior to initiation of the study. At baseline, the expert radiologists had an accuracy of 0.936 (95% CI 0.910 - 0.960) (Table 2). Radiologists had perfect specificity (1; 95% CI 0.977 - 1) and a sensitivity of 0.884 (95% CI 0.835 - 0.919). The expert-RT-PCR agreement was considered excellent (Cohen’s κ = 0.873 (0.830 - 0.920)). The accuracy of the AI for detection of any airspace opacity was 0.757 (95% CI 0.715 - 0.795). The sensitivity of the model was 0.915 (95% CI 0.872 - 0.947) and specificity was 0.586 (95% CI 0.518 - 0.652). The negative predictive value was 0.866 (95% CI 0.811 - 0.921) and indistinguishable from the expert (0.877; 95% CI 0.827 - 0.915). The interobserver agreement was considered moderate (Cohen’s κ = 0.507 (0.432 - 0.583)).

| **N = 455** | **COVID Positive (N = 235)** | | **COVID Negative (N = 220)** | |
| --- | --- | --- | --- | --- |
| **Impression** | **N** | **%** | **N** | **%** |
| No evidence of acute cardiopulmonary disease | 49 | 20.9 | 218 | 99.1 |
| Consistent with infection vs inflammatory process | 80 | 34.0 | 2 | 0.9 |
| Multifocal airspace opacities consistent with COVID-19 | 106 | 45.1 | 0 | 0 |

**Table S5**. Radiologist impressions of individual chest x-rays used in the test dataset as documented in the electronic medical record. 1 impression was unable to be retrieved.


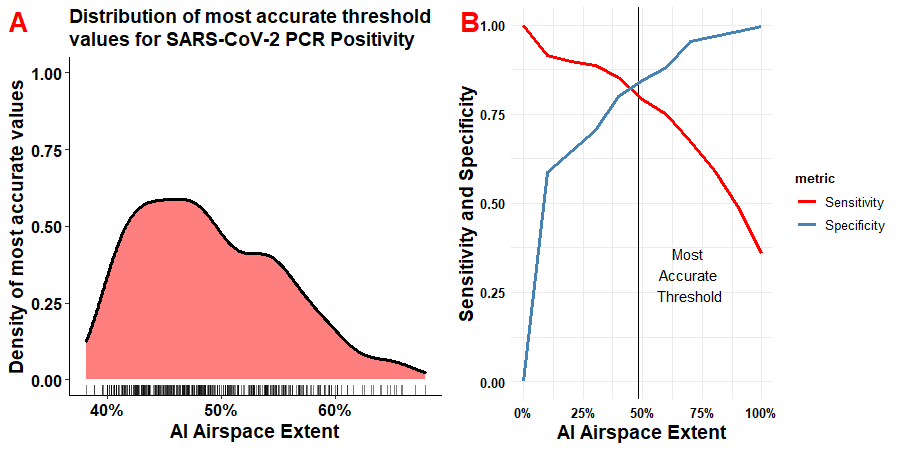


**Figure S2.** Threshold diagnostics values for SARS-CoV-2 PCR positivity using AI determined airspace extent. **A.** Distribution of most accurate threshold values using 400 bootstrapped samples. The most accurate value most likely falls between 45 - 50% AI Airspace Extent. **B.** Sensitivity and specificity trade-offs for AI Airspace Extent. The most accurate threshold is just below 50% AI Airspace Extent.
